# Effects of Deltamethrin and Endosulfan on the Hepatic Markers and Neurobehavioural Alterations in Japanese Quail (*Coturnix japonica*)

**DOI:** 10.1002/vms3.71299

**Published:** 2026-10-21

**Authors:** Saba Malik, Shoaib Ahmad, Muhammad Naeem, Iqra Bibi, Muhammad Sakandar Majid, Hafiza Sadia, Sheeba Riaz, Md Abu Bakkar Siddik

**Affiliations:** ^1^ Department of Zoology, Wildlife & Fisheries University of Agriculture Faisalabad Pakistan; ^2^ Department of Biological & Chemical Sciences University of Rasul Mandi Bahauddin Punjab Pakistan; ^3^ College of Biological Resources and Food Engineering Qujing Normal University Qujing China; ^4^ State Key Laboratory of Pharmaceutical Biotechnology NJU Advanced Institute for Life Sciences (NAILS) School of Life Sciences Nanjing University Nanjing China; ^5^ Department of Development Studies Daffodil International University Dhaka Bangladesh; ^6^ School of the Environment Nanjing University Nanjing China

**Keywords:** behavioural disruptions, deltamethrin, endosulfan, hepatic markers, lipid profile, toxicological effects

## Abstract

**Background:**

Deltamethrin (DLM), a synthetic pyrethroid, and endosulfan, an organochlorine pesticide, frequently co‐occur in agricultural environments, yet their combined effects on hepatic function and behaviour in non‐target avian species remain poorly understood.

**Objectives:**

This study examined the toxic effects of combined DLM and endosulfan exposure on hepatic markers, lipid profile and neurobehaviour in Japanese quail (*Coturnix japonica*).

**Methods:**

Twelve adult quails were divided into three groups receiving: (1) control, (2) low‐dose (0.1 mg/kg DLM + 0.15 mg/kg endosulfan) or (3) high‐dose (0.4 mg/kg DLM + 0.5 mg/kg endosulfan) via daily oral gavage for 3 weeks. Body and organ weights, serum hepatic and lipid biomarkers and behavioural parameters were assessed.

**Results:**

The high‐dose group showed a significant reduction in body weight compared with controls, while liver weight remained stable and kidney weights decreased in a dose‐dependent manner. AST (aspartate aminotransferase) and ALP (alkaline phosphatase) were substantially increased in treated groups (*p* < 0.05), indicating hepatocellular damage. Lipid profiles showed significant elevations in LDL, VLDL and cholesterol (*p* < 0.05). Behavioural assessments demonstrated dose‐dependent effects, including increased agonistic behaviour, reduced locomotor activity and dose‐dependent onset of mucoid faeces (first recorded in Week 1 in the high‐dose group and Week 2 in the low‐dose group, with none observed in controls); foraging behaviour declined sharply in high‐dose birds by Week 3.

**Conclusions:**

Combined pesticide exposure induces measurable hepatotoxicity, metabolic disruption and neurobehavioural abnormalities in quail at the tested doses. These effects are consistent with DLM‐mediated activation of Na+ channels and endosulfan‐induced blockade of GABA_A receptors.

## Graphical abstract

Japanese quail were exposed to combined low or high doses of deltamethrin and endosulfan by daily oral gavage for 3 weeks. Co‐exposure produced dose‐dependent hepatotoxicity and metabolic disruption, with elevated AST (aspartate aminotransferase), ALP (alkaline phosphatase), LDL, VLDL and cholesterol alongside reduced body and kidney weights. Treated birds also displayed neurobehavioural changes—increased aggression and reduced locomotor and foraging activity—consistent with sodium‐channel activation and GABA_A‐receptor blockade.

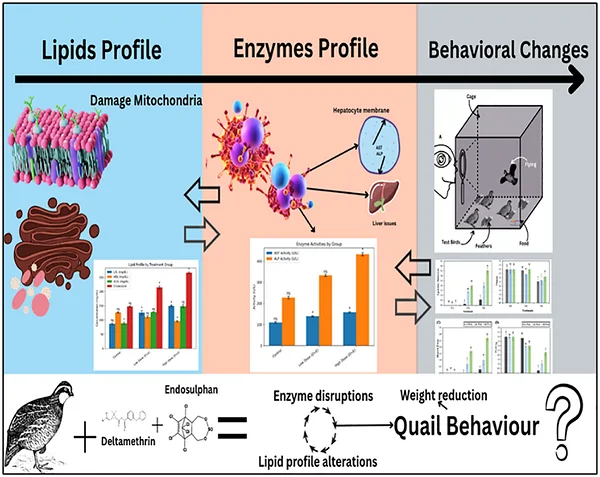

## 1 Introduction

Pesticides are used to increase crop yields (Karthick Rajan et al. 2024). However, they can enter the food chain through absorption by crops (Dhiman et al. 2024) and animals (Lu et al. 2019), ultimately posing health risks to humans (Montaigu and Goulson 2020). Deltamethrin (DLM), a synthetic pyrethroid pesticide developed in 1974, is widely utilised in agriculture for pest control (Tang et al. 2018). However, its extensive use has raised significant health and environmental concerns (Li et al. 2021). DLM residues persist in aquatic systems and soil, leading to toxic contamination and adverse effects on ecological systems (Deng et al. 2020). DLM exposure has been associated with damage to vital organs, including the liver, kidneys, nervous system and reproductive system, as well as immunotoxicity and behavioural disturbances (Robea et al. 2017). Additionally, DLM exposure may cause reductions in body and organ weight, highlighting its systemic toxicity. Given these risks, it is critical to investigate the biological and behavioural consequences of DLM exposure, particularly its impact on hepatic function and neurobehavioural outcomes in non‐target organisms.

Similarly, endosulfan (6,7,8,9,10,10‐hexachloro‐1,5,5a,6,9,9a‐hexahydro‐6,9‐methano‐2,4,3‐benzodioxathiepine 3‐oxide), an organochlorine pesticide (OCP), is classified as a persistent organic pollutant (POP) due to its environmental persistence (Sathishkumar et al. 2021) and bioaccumulative properties (Bhanot and Pant 2025). Despite its historical use in pest management, endosulfan has been globally banned due to its severe ecological impacts. Its isomers (alpha and beta, in a 7:3 ratio) have been detected in air (Yu et al. 2019), soil (Sun et al. 2017), water (Shah et al. 2023) and biota (Fang et al. 2018), demonstrating its persistence and capacity for biomagnification (Da Cuña et al. 2020). Endosulfan showed significant toxic effects on the liver, nervous system, kidneys, hematopoietic system and reproductive system (Hussain et al. 2020) as well as immunotoxicity and behavioural disturbances. Notably, its toxicity can be potentiated through combined interactions with other chemicals, exacerbating risks to ecosystems and human health (Rani et al. 2021). Therefore, it is important to investigate the biological and toxicological effects of endosulfan exposure. Figure 1 provides a detailed illustration of the processes of bioaccumulation, bioconcentration and biomagnification of residues of endosulfan and DLM, as well as their potential ecotoxicological impacts on ecosystems.

**FIGURE 1:**
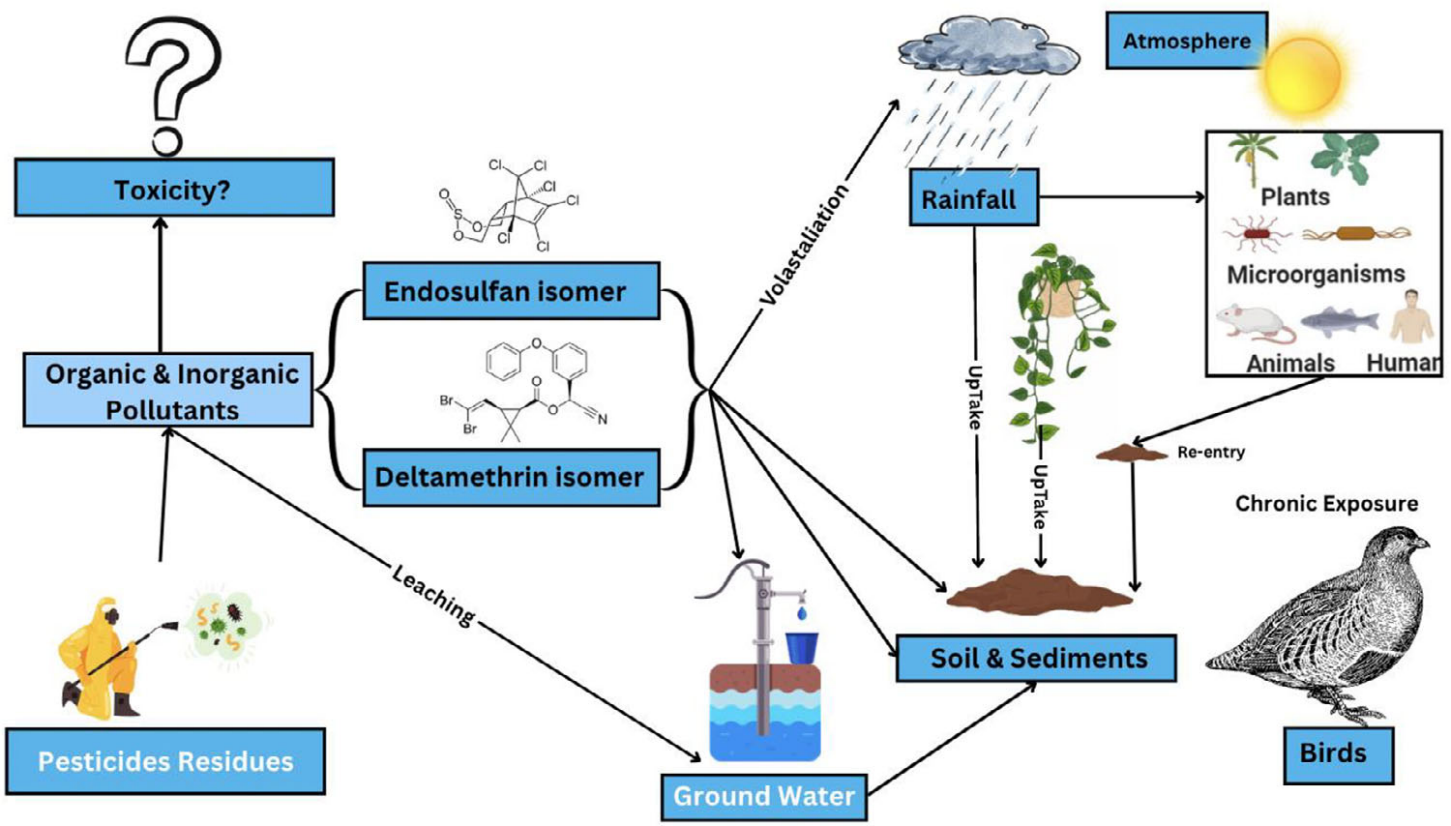
Distribution and biomagnification of endosulfan and DLM residues in the ecosystem.

Human endosulfan exposure has been documented globally (Milesi et al. 2020): one fatal suicide case in Greece (Coutselinis et al. 1978); 18 cases in India (Chugh et al. 1998); contaminated food in Turkey (Oktay et al. 2003); detection in 200 women in Spain (Botella et al. 2004); 220 young males in Spain (Carreño et al. 2007); empty endosulfan cans in India (Gude and Bansal 2012); 23 cases in Turkey (Karatas et al. 2006); 15.7% of poisoning cases in Turkey (Kır et al. 2013); 57.1% of poisonings in Iran (Soltaninejad et al. 2007); endosulfan‐related deaths in China (Zhang et al. 2013); 76.5% of children in Mexico (Flores‐Ramirez et al. 2017); and 96.5% of newborns in Spain (Monteagudo et al. 2016).

Human DLM exposure has been documented globally: 325 confirmed acute poisoning cases with seven deaths in China (He et al. 1989); 44 exposed cotton field workers in China (Gautam et al. 2025); detectable breast milk residues in Iraq (Al‐Zahra and Ahmed 2018); median daily intake of 34.3 ng/kg bw/day in France and 1.9 ng/kg bw/day in the United States (Quindroit et al. 2021); 42 poisoned patients with 78.6% mortality in India (Alam et al. 2017); a fatal intentional poisoning case in Portugal (Karasu‐Minareci et al. 2012); a suicide attempt with neurological impairment in Slovakia (Karasu‐Minareci et al. 2012); biomonitoring in human blood at 2–79 µM globally (Azmi and Naqvi 2011); a suicide case with 2.40 mg/L blood DLM in Greece (Boumba et al. 2017); and biomonitoring residues without poisoning counts in France and the United Kingdom (Shams et al. 2012).

Despite extensive research on the individual toxic effects of endosulfan and DLM, particularly regarding behavioural alterations, hepatic biomarkers and neurotoxicity, there remains a significant gap in understanding their combined impact. Notably, both pesticides are frequently used in the same agricultural regions (Sah et al. 2020) and can co‐occur in soil, water and food chains due to runoff and overlapping application cycles (Habib et al. 2024). This co‐occurrence increases the likelihood of simultaneous exposure for non‐target wildlife, including birds. Moreover, both compounds share mechanistic pathways that target the nervous system. Critically, meta‐analytic evidence indicates that co‐exposure to multiple agrochemicals at field‐realistic concentrations can interact in a greater‐than‐additive manner, amplifying toxicity beyond that predicted from single compounds (Siviter et al. 2021). DLM disrupts voltage‐gated sodium channels, while endosulfan antagonizes GABA_A receptors, both of which lead to neural hyperexcitation and potential combined neurotoxicity. Additionally, both disrupt hepatic metabolism via oxidative stress and membrane damage, potentially amplifying liver dysfunction during co‐exposure. Therefore, this study aims to evaluate the effects of combined exposure to DLM and endosulfan on Japanese quails (*Coturnix japonica*), focusing on hepatic markers, lipid profile, behavioural disruptions and changes in body and organ weights. While individual toxicity in various species of rats (Gasmi et al. 2017), rabbits (Jan et al. 2017), fish (Islam et al. 2017), chickens (Liu et al. 2022), cows (Sathishkumar et al. 2021) and humans (Qin et al. 2017) has been established, the interactive effects of DLM and endosulfan in avian species remain poorly understood. This research addresses that critical gap by investigating their combined toxicodynamics in a controlled model.

The quail (*Coturnix* spp.) has emerged as a prominent and versatile experimental model in biological research, particularly for assessing pesticide toxicity and the safety of agricultural products, owing to its rapid developmental cycle and physiological adaptability (Lukanov and Pavlova 2020; Alagawany et al. 2017). In addition to its role in scientific studies, quail is a significant food source for humans, with its breeding population steadily increasing due to desirable attributes such as high productivity, rapid growth, low production costs and culinary appeal (Redoy et al. 2017). However, the accumulation of DLM and endosulfan residues in quail tissues poses a potential risk to human health, raising concerns about food safety (Osman et al. 2023).

Classified as “Least Concern” on the International Union for Conservation of Nature (IUCN) Red List (BirdLife International 2018), quail is widely regarded as an ideal model organism for experimental research due to its ease of husbandry, disease resistance and cost‐effectiveness (Oke et al. 2025). Quails are increasingly recognised as a valuable model in experimental research due to their compact size, rapid growth, short reproductive cycle and well‐documented physiological traits. Their pronounced sensitivity to environmental changes, combined with ease of handling, makes them particularly suitable for evaluating the biological effects of environmental toxicants (Zhou et al. 2025). Economically, quails also hold significant importance in the poultry sector, being widely appreciated for their high production efficiency and favourable nutritional profile, which supports their growing demand worldwide. Nonetheless, concerns have been raised about the accumulation of pesticide residues in quail tissues, which could pose risks not only to animal health but also to human consumers through the food chain. Despite this, the effects of concurrent pesticide exposures in quails remain poorly understood, especially regarding their impact on behavioural patterns, metabolic functions and organ physiology. In this context, examining how mixed exposure to commonly used insecticides affects neurobehavioural and biochemical outcomes may provide essential insights into the broader implications of these environmental stressors for avian species and food safety.

Given these factors, it is critically important to investigate the hepatotoxic effects of DLM and endosulfan on quail liver and to evaluate associated behavioural responses to better understand the ecological and health implications of these pesticides. Endosulfan exposure in quails led to various behavioural alterations, including moderate sadness, excitement, reduced feed attraction and the presence of foamy material in faeces (mucoid faeces) (Sands et al. 2018), along with a decrease in body and organ weight, possibly due to reduced feed intake or general toxicity. Endosulfan also caused significant hepatic damage (Yan et al. 2018), affecting liver tissue (Beceren et al. 2017), metabolic processes (Katagi and Fujisawa 2021) and lipid profiles in different organisms, including quails, rats, fish and chicks. It is plausible that this damage led to increased reactive oxygen species (ROS) levels, hyperlipidemia and altered liver enzyme levels, indicating severe hepatotoxicity.

Intensive agriculture often applies DLM and endosulfan together, yet most toxicological studies examine these pesticides in isolation; consequently, we do not know how their combined presence affects wildlife, nor how biochemical injury translates into visible behavioural and physiological harm. To close this gap, our study will expose *Coturnix japonica* to environmentally relevant mixtures of the two compounds and measure (i) changes in behaviour and organ weights and (ii) the linkage between those changes and hepatic enzymes, lipid profiles and body‐weight dynamics. Clarifying these cause‐and‐effect pathways will provide a mechanistic basis for ecological‐risk assessments and help regulators set safer limits or reject binary pesticide formulations that threaten non‐target bird populations and the ecosystems they inhabit.

## 2 Materials and Methods

### 2.1 Japanese Quails and Experimental Setup

Japanese quails (*Coturnix japonica*), 12 weeks of age and weighing between 80 and 90 g, were used in this study. They were kept at 20–22°C temperature, under a 12 h light/12 h dark cycle and at 15% humidity for acclimatisation. The overall cage size is 60 cm in length, 50 cm in height and 40 cm in depth. The cage is divided into two equal sections, with each section measuring 30 cm in length, 50 cm in height and 40 cm in depth (Figure 2). The animal experiments were performed in accordance with the guidelines of the University of Agriculture, Faisalabad, Pakistan. Japanese quails (*Coturnix japonica*) were acclimatised for 1 week before the experimentation. During the acclimatisation period, the birds were provided with normal feed and drinking water daily in the morning (8:30 AM), afternoon (2:30 PM) and evening (7 PM). The cages were sanitised three times a week using a freshly prepared 10% sodium hypochlorite solution. Efforts were made to minimise external disturbances such as slamming doors, loud conversations in the experimental room and strong external odours (e.g., perfumes). These measures aimed to reduce stress‐inducing environmental stimuli that could influence the animals' behaviour, thereby preventing undesirable actions such as feather pecking, aggression, social abnormalities and signs of distress, including starvation, feather damage and lethargy. After adaptation/acclimatisation for 1 week, 12 Japanese quails (*Coturnix japonica*) were split into three groups (4 birds in each group). Group 1 was named the control group; Group 2 was named the low‐dose group; and Group 3 was named the high‐dose group. However, all groups had four quails. Daily, birds were provided with fresh feed and pure water.

**FIGURE 2:**
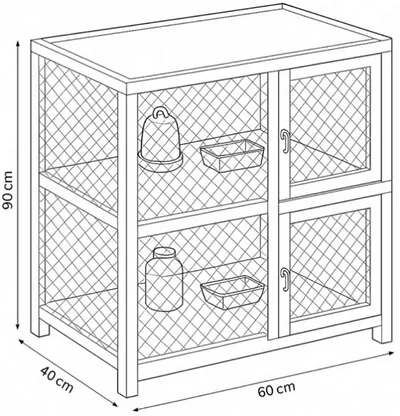
Views of the apparatus used during the open field test performed on *Coturnix japonica*.

### 2.2 Experimental Period and Treatment

The treatment groups received the insecticide mixture (DLM + endosulfan) for 3 weeks following acclimatisation and grouping. Insecticides were purchased from Sigma‐Aldrich (Germany). Oral gavage was selected as the route of administration for all treatments. It is the most widely used administration route in animal studies (Taylor et al. 2023; Bommarito et al. 2026). It provides precise control over dose volume, timing and delivery directly to the stomach (Shrestha and Préat 2020). It is also a standard method in non‐human primate research (Harvey 2023). The first group served as the control group, the second group was supplied with a low dose of (DLM + endosulfan) and the third group was treated with a high dose of (DLM + endosulfan). The detailed justification for the selected doses (Table 1) is provided in Supporting Information.

**TABLE 1:** An experimental diet was fed to quails during the experimental period.

Stages/time periods	Weeks	Requirement (mg/kg) per day
Acclimatization period	1	Fresh feed and pure water
Control group treatment	3	Fresh feed and pure water
Low dose treatment period	3	Fresh feed, pure water, 0.1 mg DLM + 0.15 mg endosulfan
High dose treatment period	3	Fresh feed, pure water, 0.4 mg DLM + 0.5 mg endosulfan

### 2.3 Test Diet Preparation

DLM and endosulfan (98% purity) were procured from Sigma‐Aldrich, Germany. For the preparation of the dose formulations, 100 g of each insecticide was dissolved in 100 mL of distilled water, with 0.7 mL of ethanol added to enhance solubility. Details of the dose formulations for DLM and endosulfan, including the high and low doses administered over the 3‐week trial period, are presented in Supporting Information.

### 2.4 Behavioural Tests

Japanese quails were exposed to a series of behavioural assessments (Figure 3) based on the hypothesis that ingestion of endosulfan and DLM may impair the central nervous system of non‐target organisms. Consequently, indicators of neurological dysfunction, such as vocalisation patterns, reduced feed attraction, physical activity levels and faecal characteristics, were monitored and qualitatively assessed daily following pesticide administration. Each observed clinical sign was assigned a score ranging from 0 to +4, reflecting its intensity and frequency (0 = absent, +1 = minimal, +2 = mild, +3 = moderate and +4 = severe).

**FIGURE 3:**
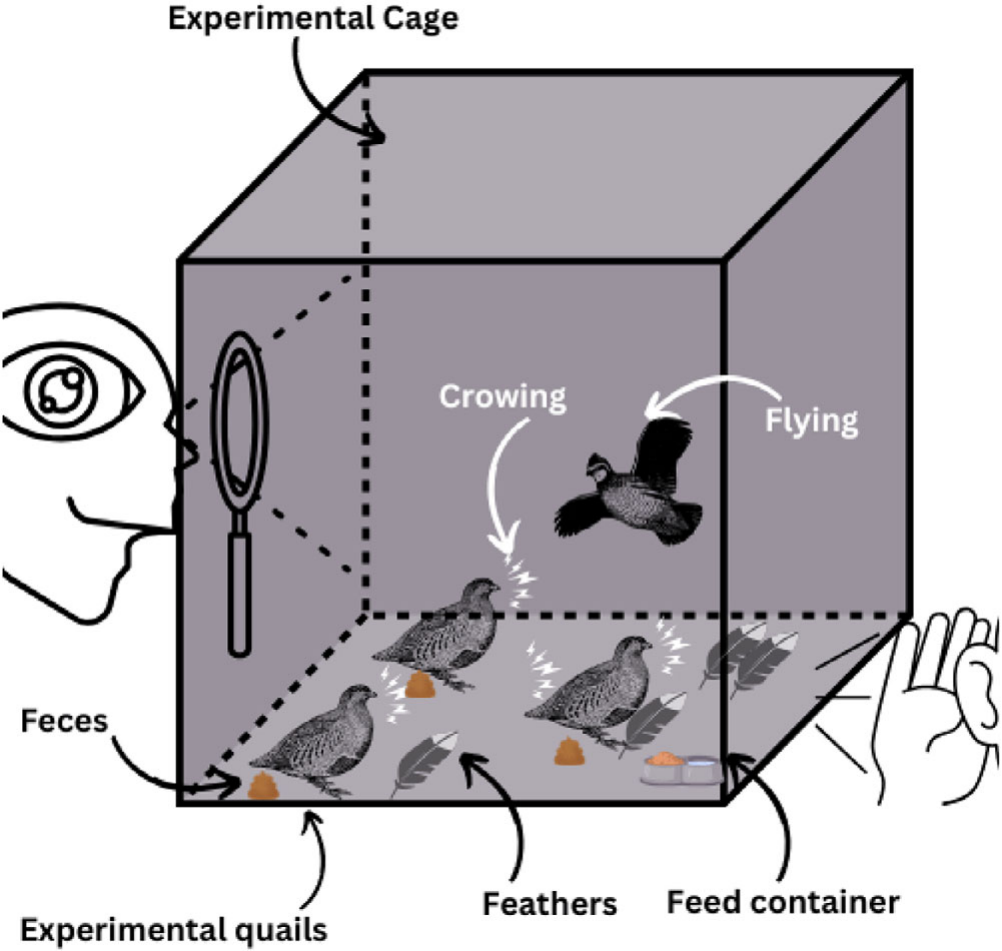
Illustrative figure of behaviour experiments: agonistic behaviour test, locomotor activity, mucoid faeces test and foraging behaviour.

Under natural conditions, agonistic behaviours in quails predominantly involve pecking and vocalisations (crowing), with physical confrontations occurring less frequently. Within a group hierarchy, the dominant male typically exerts control over subordinate males, restricting their access to resources such as food through displays of territoriality and social dominance.

### 2.5 Blood and Organ Collections

On the final day of the experimental period (at the end of the 3rd week), blood samples were collected from each bird immediately following euthanasia. Serum was separated from the blood and stored at −20°C to ensure preservation for subsequent biochemical analyses. Birds were first exposed to ethanol. Following anaesthesia, birds were humanely euthanised in accordance with the AVMA Guidelines for the Euthanasia of Animals (AVMA 2020) and exsanguinated through a thoracic incision to achieve maximal blood drainage. The kidneys and liver were carefully dissected, cleaned of surrounding fat, gently dried and weighed separately. Blood samples were collected from the quails, placed into test tubes using a funnel and stored at −20°C for subsequent biochemical analysis.

### 2.6 Biochemical Analysis

Enzyme concentrations were measured using a commercially available Aspartate Assay Kit, adhering strictly to the manufacturer's protocol. The lipid profile, comprising low‐density lipoprotein (LDL), very‐low‐density lipoprotein (VLDL) and total cholesterol, was analysed using a validated Cholesterol Assay Kit, following established analytical procedures.

### 2.7 Statistical Analysis

Statistical analysis was conducted using one‐way analysis of variance (ANOVA), followed by Tukey's post hoc test, to evaluate differences between control and experimental groups. Statistical significance was defined at *p* < 0.05. Data for behavioural changes, body weight and organ weight are presented as mean ± standard deviation (SD). Data for hepatic damage markers and lipid profile are presented as mean ± standard error of the mean (SEM). All analyses were performed in Python using the SciPy library (Virtanen et al. 2020) on the Windows operating system.

### 2.8 Ethical Consideration

The experimental procedures conducted in this study were reviewed and approved by the Ethics Committee of the Department of Zoology, Wildlife, and Fisheries at the University of Agriculture, Faisalabad, ensuring compliance with ethical standards for research involving animals.

## 3 Results

### 3.1 The Effect of DLM and Endosulfan Exposure on Body Weight and Organ Weight of Quail

The control group maintained slightly higher body weights than the low‐dose and high‐dose groups, particularly during the early weeks of the trial (Week 1 to Week 3). Moreover, the body weight showed a dose‐dependent decrease in the DLM and endosulfan treatment groups across the experimental period (Figure 4A). The liver of the quail remained stable over the entire observation period, as indicated by the standard deviation (Figure 4B). In contrast, the kidney size varied significantly. The dose‐dependent decline in quail organ weights indicates subclinical organ toxicity and early‐stage physiological alterations resulting from exposure to DLM and endosulfan. Importantly, no mortality was recorded in any treatment group throughout the study.

**FIGURE 4:**
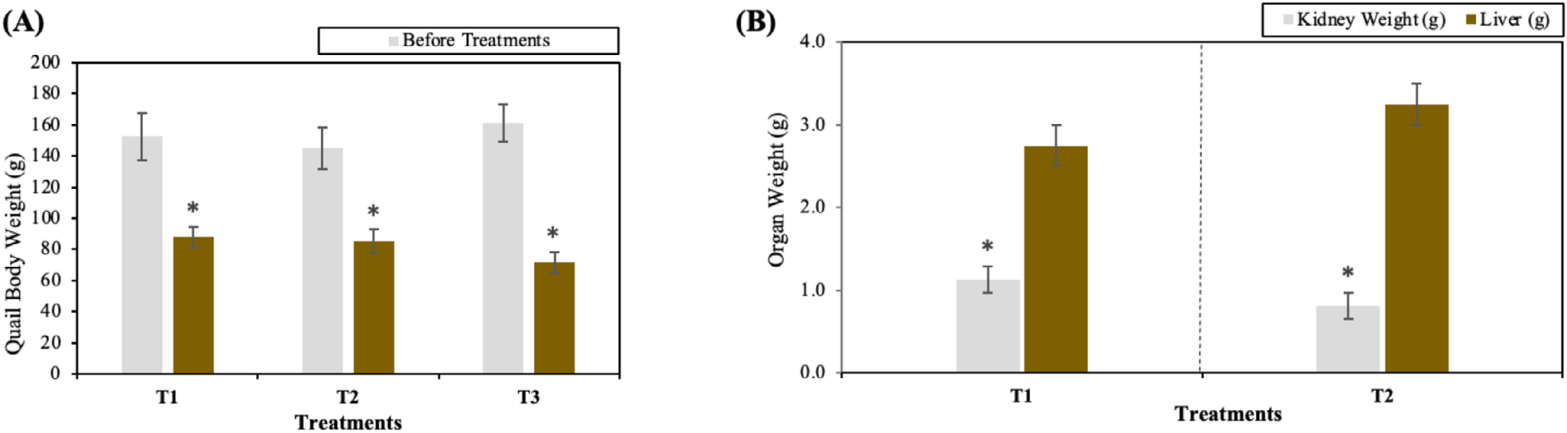
DLM and endosulfan induced body and organ weight changes in quails. (A) Quail body weight changes control group (T1), low dose (T2) and high dose (T3). (B) Quail organ weight changes, low dose (T1) and high dose (T2). Data are presented as mean ± SD, *n* = 4. *p – statistical significance.

### 3.2 Behavioural Changes

Signs observed immediately after treatment included: agonistic behaviour, mucoid faeces, foraging and locomotor activity. Observations were recorded daily across the 3‐week treatment period. Other observations at this stage included reduced activity, anorexia, dullness, aggression, head nutation, increased excitement, attempts to fly and whole‐body shivering. Agonistic behaviour showed a dose‐dependent increase in activity under DLM and endosulfan treatment (Figure 5A). The locomotor activity graph (Figure 5B) shows that the control group maintained a stable locomotor activity level of 4 throughout the trial, while the low‐dose group exhibited fluctuations, and the high‐dose group showed a marked decrease, indicating declining physical health with increased dosage. The mucoid faeces behaviour further highlights the adverse effects of the insecticide. The control group maintained consistent foraging behaviour and showed no mucoid faeces at any point during the trial. Mucoid faeces frequency increased in both dosed groups in a dose‐dependent manner, with frothy material appearing first in the high‐dose group during Week 1 and in the low‐dose group during Week 2, and increasing further in both groups by Week 3, indicating worsening gastrointestinal distress (Figure 5C). This suggests systemic toxicity and compromised gut function. The low‐ and high‐dose groups exhibited reduced foraging behaviour, with the high‐dose group showing a sharp decline by Week 3, indicating severe anorexia (Figure 5D), which indicates severe anorexia and reduced energy intake likely associated with metabolic stress or neuroinflammation. These behavioural effects are associated with underlying biochemical and physiological stress induced by exposure to DLM and endosulfan.

**FIGURE 5:**
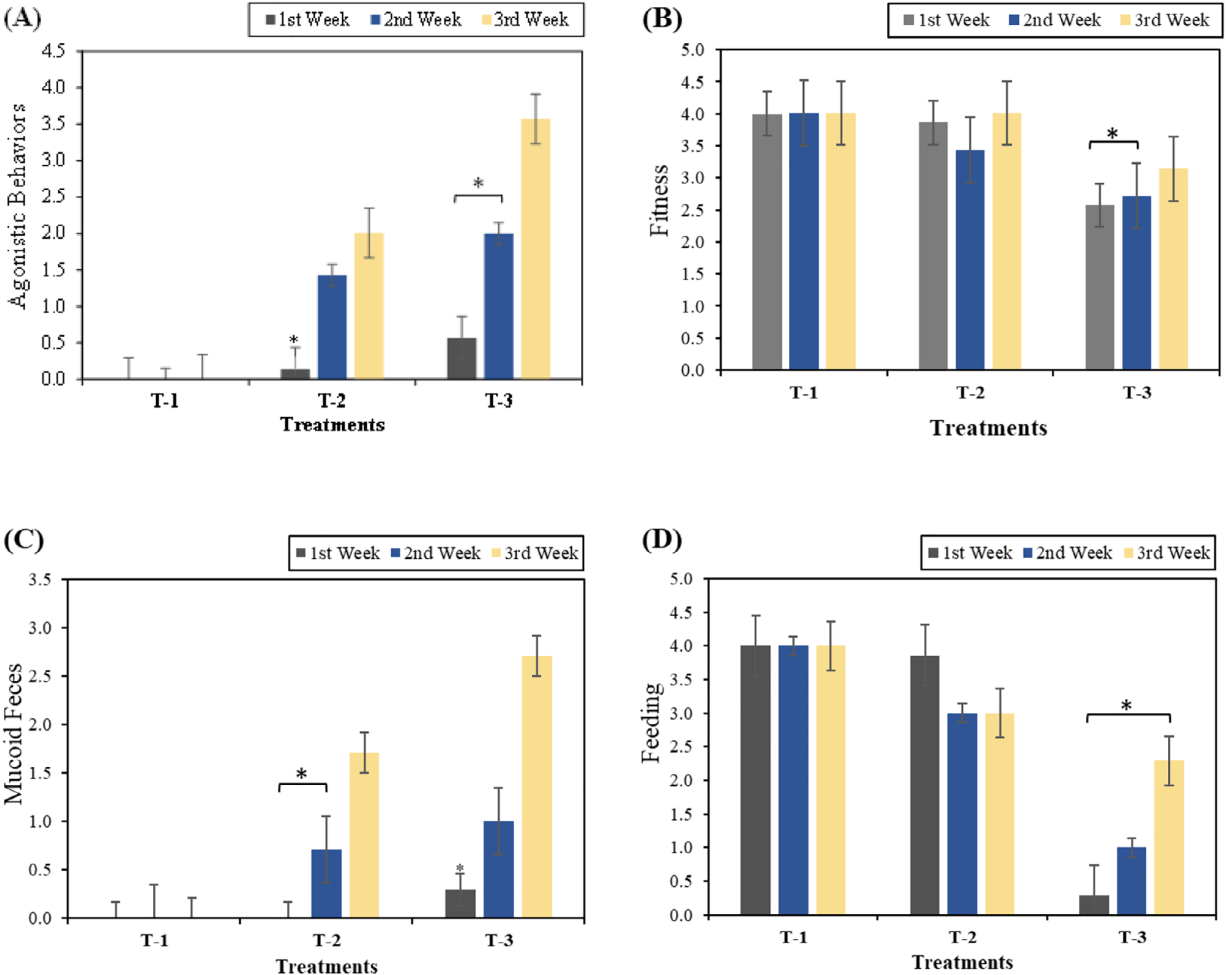
DLM and endosulfan‐induced behaviour changes in quails: (A) Agonistic behaviour. (B) Locomotor activity. (C) Mucoid faeces. (D) Foraging behaviour. Data are presented as mean ± SD, *n* = 4. *Statistically different (*p* < 0.05); control group (T1), low dose (T2) and high dose (T3).

### 3.3 DLM and Endosulfan Increased the Levels of Hepatic Damage Markers

Hepatic damage markers AST (aspartate aminotransferase) and ALP (alkaline phosphatase) in the liver were determined. Compared with the control group, ALP and AST levels were significantly increased in both the low‐dose and high‐dose groups (Figure 6). Significant changes occurred in the low‐dose (0.1 mg/kg BW DLM + 0.15 mg/kg BW endosulfan) and high‐dose (0.4 mg/kg BW DLM + 0.5 mg/kg BW) experimental groups (*p* < 0.05).

**FIGURE 6:**
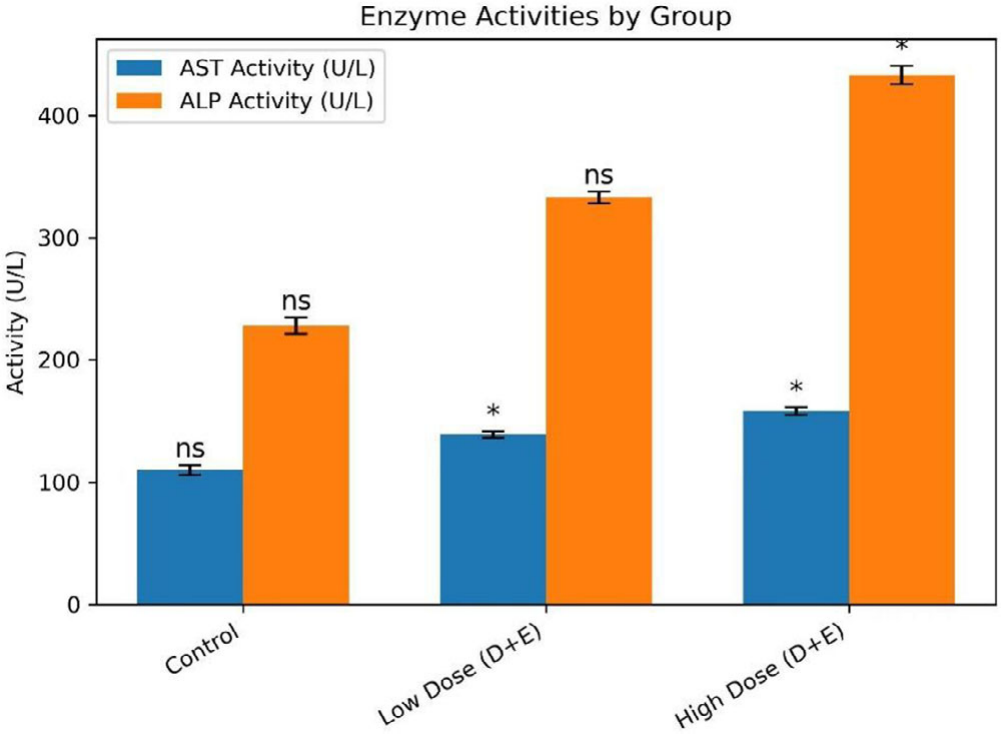
Effects of DLM and endosulfan treatment on the biomarker levels (AST activity and ALP activity in quails). Data are presented as mean ± SEM (*n = 3*). * Notably different (*p* < 0.05) versus control group.

### 3.4 DLM and Endosulfan Induced Substantially Increased Levels of Lipid Profile

LDL, VLDL and cholesterol are indicators of hepatic damage. Endosulfan and DLM toxicity show increased levels of LDL, VLDL and cholesterol in the liver of quails (Figure 7). The effect of the insecticide mixture (endosulfan + DLM) on the level of LDL, HDL, VLDL and cholesterol in control and treated groups of quails shows a significant (*p <* 0.05) difference after 3 weeks of treatment.

**FIGURE 7:**
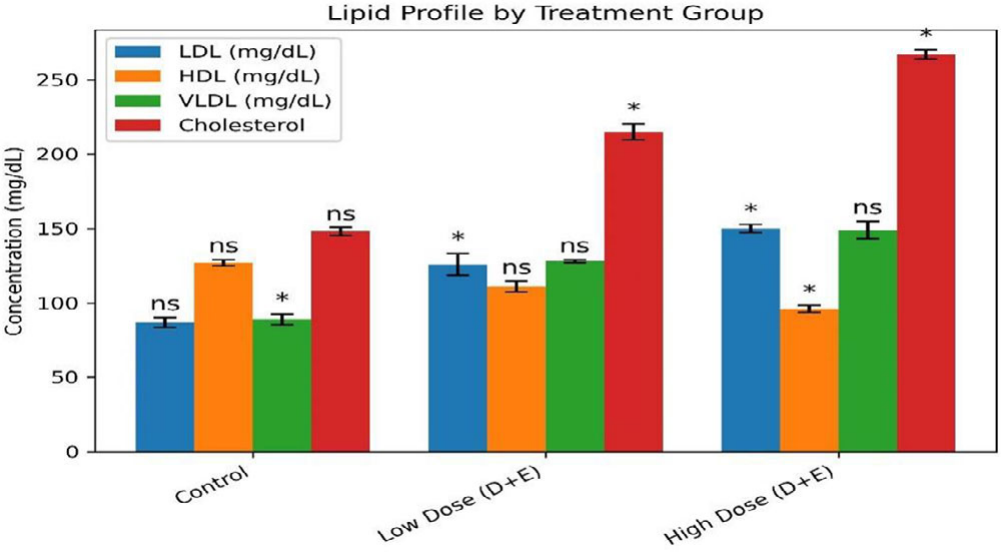
Effects of DLM and endosulfan treatment on the biomarker levels (LDL level, cholesterol level and VLDL level) in quails. Data are presented as mean ± SEM (*n* = 3). *Notably different (*p* < 0.05) versus control group.

## 4 Discussion

Food security remains a pressing concern in many low‐income countries, where the extensive use of pesticides such as endosulfan and DLM poses serious environmental and health risks (Tambo et al. 2024). Endosulfan has been reported to cause toxicity in both domestic and wild birds through ingestion of contaminated food sources (Menezes et al. 2017). Similarly, the widespread application of DLM as a synthetic pyrethroid insecticide has raised significant concerns due to its documented environmental impacts (Shi et al. 2024) and potential health hazards to humans (Rehman et al. 2017). While previous studies have primarily focused on the individual toxicity of these compounds, the present study is the first to demonstrate dose‐dependent physiological and behavioural alterations in quails resulting from combined exposure. Specifically, co‐exposure to DLM and endosulfan resulted in measurable changes in behaviour and organ weights, as well as marked disruptions in hepatic enzyme activity and lipid metabolism. These biochemical alterations may be associated with oxidative stress, organ atrophy and signs of neurotoxicity, ultimately resulting in body weight reduction and impaired behavioural performance in quails.

The significant increases in AST and ALP levels observed in both treatment groups suggest hepatic injury and support pesticide‐induced hepatotoxicity. These biochemical alterations correspond with the dose‐dependent reductions in liver and kidney weights, further indicating organ‐specific toxicity. Additionally, the marked elevations in LDL, VLDL and total cholesterol levels indicate disruptions in lipid metabolism, often associated with hepatic inflammation and impaired liver function. Although overall body weight changes were not statistically significant, the reduction in vital organ weights points to physiological stress. Behavioural changes, particularly reduced foraging activity, increased vocalisation and decreased physical performance in the high‐dose group, indicate potential neurotoxic effects, which could involve oxidative stress and inflammatory pathways, though oxidative stress markers were not directly measured in this study. Taken together, these results demonstrate a clear relationship between hepatic enzyme disruption, altered lipid profiles, organ weight loss and behavioural abnormalities in pesticide‐exposed quails, thus addressing and supporting the research question.

The dose‐dependent reductions in liver and kidney weights observed here merit particular attention, since organ enlargement is sometimes assumed to accompany toxicant exposure. In birds, however, chronic pesticide exposure more commonly reduces relative organ weights through suppressed feed intake, impaired anabolic protein synthesis and parenchymal atrophy or necrosis rather than inflammatory hypertrophy. This interpretation is consistent with Qamar et al. (2012), who administered endosulfan alone to male Japanese quail (*Coturnix japonica*) and reported significantly decreased feed intake, body weight and relative organ weights, together with hepatic fatty change and necrotic foci. Notably, in that study endosulfan produced no overt clinical signs at 5 mg/kg feed, whereas the present study elicited measurable behavioural and organ‐weight effects at substantially lower endosulfan doses (0.15–0.5 mg/kg) when co‐administered with DLM. This contrast supports a greater‐than‐additive interaction between the two compounds and offers a plausible explanation for the apparent discrepancy with single‐compound studies: Combined exposure appears to lower the threshold at which organ‐level toxicity becomes detectable.

### 4.1 Hepatic Enzyme Disruption

Significant increases in AST and ALP (*p* < 0.05) were observed in both low‐ and high‐dose groups (Figure 6A,B) compared to the control (Figure 6A,B), confirming dose‐dependent hepatotoxicity. AST activity (U/L) and ALP activity (U/L) are non‐specific, immunocompetent enzymes (Ziyanok‐Demirtas 2024) and are indicators of hepatic damage in the liver and are useful for assessing stress in organisms (Kumar et al. 2016). Elevated plasma levels of AST and ALP are key indicators of cellular dysfunction, reflecting hepatocellular damage, necrosis, oedema, inflammation, alterations in membrane permeability and cholestasis (Liu et al. 2021). As stated by Abdel‐Daim et al. (2020), AST and ALP indicate liver injury and damage, as these enzymes serve as hepatic markers of liver dysfunction.

These results are consistent with the findings of Chaouchi et al. (2025), who reported that elevated levels of ALT and ALP are associated with hepatic cytolysis and liver injury resulting from DLM exposure. This aligns with previous research (Li et al. 2022), reinforcing the conclusion that such enzymatic changes reflect damage to hepatopancreatic cells.

Elevated AST, particularly its mitochondrial isoform, is a sensitive biomarker of extensive cellular damage and compromised mitochondrial membrane integrity (Fang et al. 2024). This observation aligns with the known hepatotoxic mechanisms of DLM, which promote ROS generation and disrupt hepatocyte membrane structures (Abdel‐Daim et al. 2020). Concurrently, endosulfan contributes to hepatocellular damage by initiating lipid peroxidation cascades (Saxena et al. 2011). The combination of these two pesticides appears to exert a combined toxic effect, as reflected by the significantly elevated serum AST and ALP levels observed in quails subjected to co‐exposure. Although prior studies have demonstrated that DLM alone is capable of inducing substantial increases in AST and alanine aminotransferase (ALT) levels in fish models (Abdel‐Daim et al. 2020), the current findings suggest that the simultaneous administration of DLM and endosulfan amplifies hepatic injury. This enhanced biochemical response indicates an interactive toxicodynamic effect, which may arise from the convergence of oxidative and membrane‐disruptive pathways (as illustrated conceptually in Figure 8).

**FIGURE 8:**
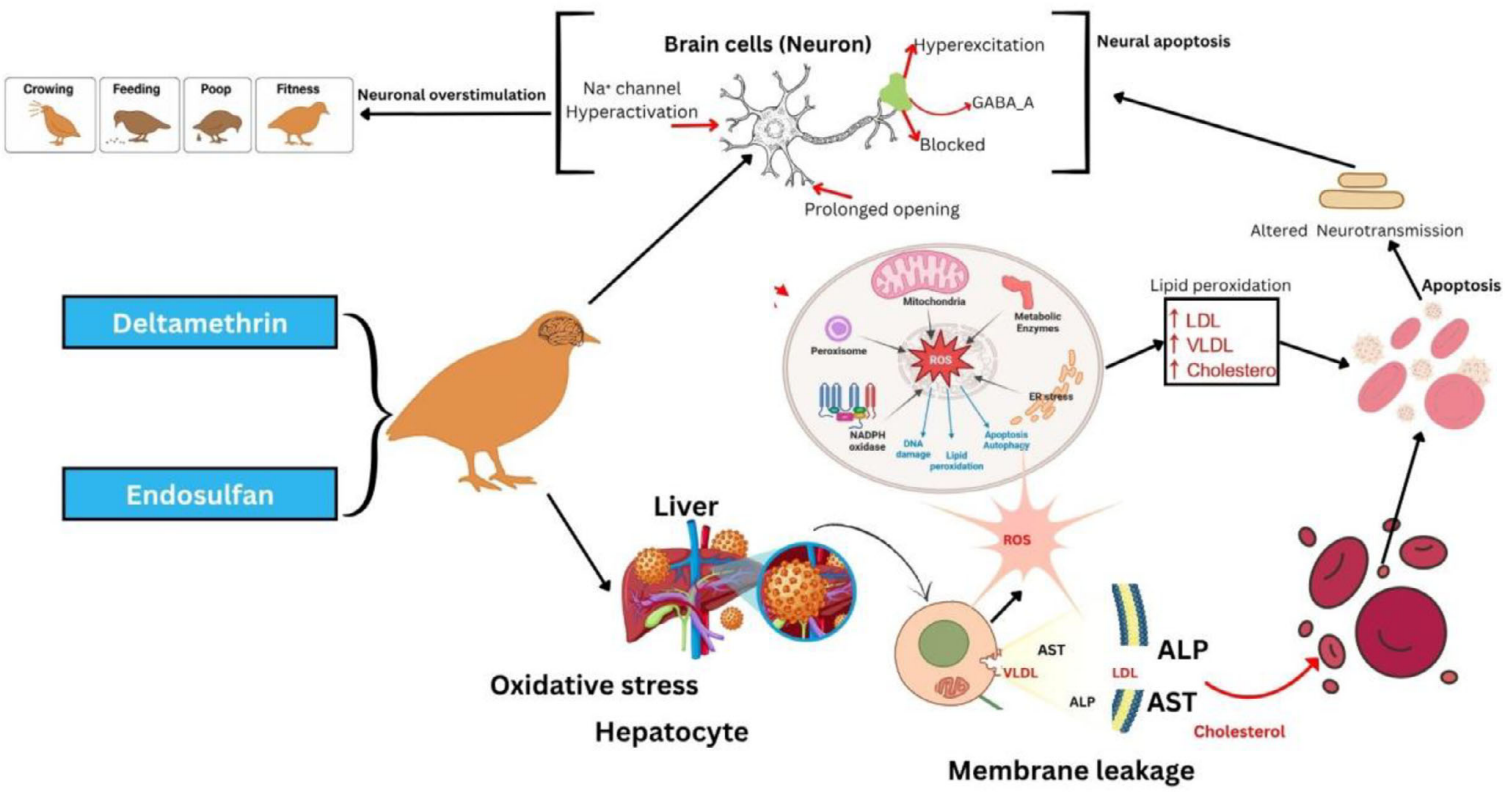
Mechanistic links between combined DLM–endosulfan exposure and quail health: Hepatic dysfunction, lipid disruption and neurobehavioural deficits in *Coturnix japonica*.

The magnitude of serum AST and ALP elevation in *Coturnix japonica* closely parallels hepatotoxic responses previously documented in rodent models (Chaouchi et al. 2025). However, the accelerated onset of enzymatic perturbations observed in this avian model suggests a comparatively higher susceptibility to combined xenobiotic exposure. This increased sensitivity is further corroborated by histopathological evidence of hepatic parenchymal atrophy in the high‐dose exposure groups, indicative of extensive hepatocellular necrosis and diminished functional hepatic mass (Li et al. 2021). Although AST and ALP serve as classical biomarkers of hepatocellular and biliary injury, respectively, quantification of lipid peroxidation (e.g., malondialdehyde [MDA]) and antioxidant defence parameters (e.g., superoxide dismutase [SOD]; catalase [CAT]; glutathione peroxidase [GPx]) is recommended in future investigations to delineate the role of redox imbalance in pesticide‐induced hepatic injury. They do not capture the redox imbalance that underlies this damage.

As shown in Figure 8, combined exposure to DLM and endosulfan disrupts membrane integrity, alters hepatic enzyme function and impairs lipid homeostasis. It is hypothesised that these effects are mediated by the increase of ROS, although ROS were not directly measured in this study. These biochemical and structural alterations result in organ dysfunction and contribute to the observed behavioural abnormalities. A limitation of this study is that oxidative stress markers were not directly measured. To validate this mechanistic model, future studies should include quantification of oxidative stress markers such as MDA, SOD and GPx.

### 4.2 Lipid Metabolism Disruption

The liver is a critical organ in lipid metabolism, playing a central role in regulating essential physiological processes (Alamri 2018). As reported by Abdelkhalek et al. (2022), pesticide‐induced toxicity is often mediated through lipid peroxidation, which arises from the overproduction of free radicals or ROS. Hepatic steatosis occurs when the balance between fatty acid uptake, synthesis, oxidation and secretion is disrupted (Yang and Park 2018). Excessive free fatty acids (FFAs) are converted into triglycerides (TGs), and their accumulation leads to lipid overload, resulting in hepatocellular steatosis (Li et al. 2022). If unregulated, this condition can progress to severe hepatic disorders such as fibrosis, cirrhosis and hepatocellular carcinoma (Nieradko‐Iwanicka and Borzęcki 2015).

Lipid toxicity, often associated with impaired lipid metabolism, is directly linked to mitochondrial dysfunction, which can trigger apoptosis (Bai and Tang 2025). Furthermore, oxidative stress exacerbates mitochondrial damage, leading to increased hepatocyte apoptosis (Fang et al. 2024). Our study supports these findings, as exposure to endosulfan and DLM significantly elevated hepatic lipid markers, including LDL, VLDL and cholesterol, in quails after 3 weeks of treatment. These alterations indicate hepatic metabolic distress, suggesting that pesticide exposure may contribute to lipid accumulation and liver dysfunction.

Previous studies have shown that mitochondrial damage plays a crucial role in the disruption of lipid metabolism and in hepatotoxicity (Oliveira et al. 2017). Consistent with this, the observed increase in LDL, VLDL and cholesterol levels in pesticide‐treated quails suggests a disturbance in lipid homeostasis, potentially mediated by mitochondrial dysfunction.

Our findings indicate that combined exposure to DLM and endosulfan disrupts hepatic lipid metabolism, as shown by elevated LDL, VLDL and cholesterol levels in Figure 8. The significant elevation in LDL, VLDL and cholesterol levels observed in treated quails suggests impaired lipid clearance and enhanced lipid accumulation, consistent with pesticide‐induced dysregulation of lipid homeostasis (Tung et al. 2017). This disruption is mechanistically linked to mitochondrial damage, which compromises β‐oxidation and promotes lipid peroxidation (Hamidipoor et al. 2016). As shown in Figure 8, these effects may be further mediated by increased ROS, leading to membrane damage, hepatic enzyme leakage (AST; ALP) and apoptosis (Li et al. 2022). Elevated lipid markers thus serve not only as indicators of metabolic distress but also as active contributors to hepatocyte dysfunction and reduced liver mass in *Coturnix japonica* (Yan et al. 2021).

### 4.3 Behavioural and Biochemical Observations

The behavioural profile of *Coturnix japonica* exposed to combined DLM and endosulfan reveals a consistent pattern of neurotoxicity and systemic physiological dysfunction, with symptoms intensifying in a dose‐dependent manner. Notably, agonistic behaviour (extreme crowing and agitation) was observed in the high‐dose group beginning on the 4th day of Week 3, often accompanied by frantic flight attempts and collisions within the cage. The low‐dose group exhibited moderate agonistic behaviour, while control birds showed no deviation from baseline behaviour. These symptoms align mechanistically with sodium channel hyperactivation and GABA_A receptor inhibition, leading to neurohyperexcitation, a hallmark of DLM and endosulfan neurotoxicity (Li et al. 2021). This mechanistic pathway is illustrated in Figure 8, which summarises prolonged Na^+^ channel opening and impaired GABAergic signalling, resulting in neuronal overstimulation and apoptosis.

Foraging behaviour was significantly suppressed in both treatment groups (*p* < 0.05), with greater reduction observed in the high‐dose group. This dose‐dependent decline in foraging is consistent with earlier findings by Kumar et al. (2017) and may be linked to disruption of hepatic lipid metabolism, as evidenced by elevated LDL, VLDL and cholesterol levels in the biochemical profile. Impaired liver function likely interfered with nutrient assimilation and energy balance, contributing to the observed foraging suppression. Alertness deficits appeared by Week 1 in the high‐dose group, marked by lethargy, poor coordination and reduced reactivity. The low‐dose group showed milder, yet significant, alertness issues. These mirror the dizziness and weakness reported in endosulfan‐treated birds (Mohamed et al. 2017), further supporting the neurotoxic mechanism. Gastrointestinal disturbances, particularly mucoid faeces, appeared in the high‐dose group during the first week and in the low‐dose group during the second week. No such symptoms were observed in controls. These signs are likely due to hepatic dysfunction impacting bile acid synthesis and digestion (Yan et al. 2018). Figure 8 links liver membrane damage and enzyme leakage (elevated AST and ALP) to systemic physiological outcomes.

Compared to prior studies, our results show both alignment and novel patterns. Behavioural anomalies resembling anxiety and reduced foraging have been reported in pesticide‐exposed quails (Crisol‐Martínez et al. 2016). However, our study observed earlier symptom onset by Week 1, unlike Deng et al. (2021), who reported effects in Week 3. This suggests a combined toxic interaction from pesticide exposure. Additionally, reproductive behaviours such as mounting and crowding indicators of sexual maturity in male quails (de Faria et al. 2018) were delayed or absent in treated birds. This may reflect endocrine disruption, as both pesticides are known hormonal disruptors in avian models. Collectively, these findings support a model of systemic toxicity in *Coturnix japonica*, where behavioural, hepatic and reproductive dysfunction are interconnected. However, a key limitation of the experimental design is the absence of groups exposed solely to DLM or solely to endosulfan. Second, histopathological evaluation of liver and kidney tissues was not conducted. Additionally, renal function markers (creatinine and urea) and the liver enzymes (ALT and GGT) were not measured. Addressing these limitations in future work—through the inclusion of single‐pesticide control groups, liver and kidney histopathology and renal (creatinine and urea) and additional hepatic (ALT and GGT) markers—would further resolve the mechanisms and organ specificity of combined DLM–endosulfan toxicity.

## 5 Conclusion

This study effectively addresses two central research questions regarding the effects of combined exposure to DLM and endosulfan in *Coturnix japonica*. First, the observed behavioural abnormalities and organ weight alterations are linked to the interaction of two neurotoxic mechanisms: prolonged neuronal excitation via DLM‐induced Na^+^ channel activation and endosulfan‐induced blockade of GABA_A receptors. These neurophysiological disruptions may be further intensified by oxidative and organellar stress, though oxidative stress markers were not directly measured. Second, the study reveals a mechanistic connection between hepatic enzyme dysregulation, altered lipid metabolism and behavioural changes. Pesticide‐induced oxidative stress not only impairs hepatocyte integrity, evidenced by elevated AST and ALP levels, but also leads to significant increases in LDL, VLDL and total cholesterol, indicating a metabolic imbalance. This biochemical stress correlates with reduced foraging and lethargy, suggesting a bidirectional relationship between metabolic dysfunction and neurobehavioural outcomes. Although no significant change in body weight was detected, internal organ damage and biochemical disruptions indicate substantial subclinical toxicity. These findings underscore the importance of evaluating mixture toxicity and call for stricter ecotoxicological assessments of pesticide combinations in avian species.

## Author Contributions

**Saba Malik**: conceptualisation, methodology, investigation, formal analysis, writing –original draft. **Shoaib Ahmad**: methodology, investigation, data curation. **Muhammad Naeem**: formal analysis, software, visualisation. **Iqra Bibi**: investigation, resources. **Muhammad Sakandar Majid**: investigation, validation. **Hafiza Sadia**: data curation, visualisation. **Sheeba Riaz**: formal analysis, writing – review and editing. **Md Abu Bakkar Siddik**: supervision, writing – review and editing.

## Funding

The authors have nothing to report.

## Ethics Statement

The experimental procedures conducted in this study were reviewed and approved by the Ethics Committee of the Department of Zoology, Wildlife, and Fisheries, University of Agriculture, Faisalabad, Pakistan. All procedures complied with the relevant national, institutional and international guidelines for the humane care and use of animals in research.

## Conflicts of Interest

The authors declare no conflicts of interest.

## Supporting information




**Supporting File 1**: vms371299‐sup‐0001‐S1.docx

## Data Availability

Data are available from the corresponding author upon reasonable request.
