# Supplementary material for: Effects of Deltamethrin and Endosulfan on the Hepatic Markers and Neurobehavioural Alterations in Japanese Quail (Coturnix japonica)
Source: Vet Med Sci. 2026 Oct 21;12(6):e71299. doi: 10.1002/vms3.71299 (PMC13685525; doi:10.1002/vms3.71299)
Supplement: Supplementary file 1 [file VMS3-12-e71299-s001.docx]

**Supporting Information for**

**Effects of Deltamethrin and Endosulfan on the hepatic markers and neurobehavioral alterations in Japanese quail (*Coturnix japonica*)**

#### Saba Malik^a^, Shoaib Ahmad^b^, Muhammad Naeem^c^, Iqra Bibi^a^, Muhammad Sakandar Majid^a^, Hafiza Sadia^d^, Sheeba Riaz^d^, Md Abu Bakkar Siddik^*^

*^a^Department of Zoology, Wildlife & Fisheries, University of Agriculture, Faisalabad, Pakistan.*

*^b^Department of Environmental Sciences, Government College University Faisalabad, Faisalabad 38000, Pakistan.*

*^b^College of Biological Resources and Food Engineering, Qujing Normal University, Qujing, China.*

*^d^State Key Laboratory of Pharmaceutical Biotechnology, NJU Advanced Institute for Life Sciences (NAILS), School of Life Sciences, Nanjing University, Nanjing 210023, China.*

**Corresponding author E-mail address:* rmksiddik@gmail.com

**The selection of Endosulfan and deltamethrin concentrations.** Endosulfan and deltamethrin concentrations in environmental matrices reported in the public literature range widely. For endosulfan, soil concentrations range from 0.013 to 0.136 mg/kg (Oh et al., 2020), while surface water concentrations reach 37.56 µg/L (Hashmi et al., 2019). For deltamethrin, groundwater concentrations range from 0.3 to 0.7 µg/L, agricultural wastewater contains up to 11.62 µg/L, and soil residues are approximately 0.01 mg/kg shortly after spraying (Zardosht et al., 2023). Background levels in unpolluted areas are typically near or below detection, whereas agricultural runoff and irrigation return flows show higher concentrations. Therefore, the low dose (0.1 mg/kg DLM + 0.15 mg/kg endosulfan) and high dose (0.4 mg/kg DLM + 0.5 mg/kg endosulfan) used in this study represent environmentally relevant sub‑lethal exposure scenarios for birds feeding in contaminated habitats.

Our dose selection is further supported by published toxicological evidence indicating sub‑lethal exposure levels for quails and other poultry species (Denny and Stewart, 2013). Several studies in quail have used chronic oral DLM doses ranging from 15–45 mg/kg body weight to induce measurable but non‑lethal biochemical and physiological effects, including brain apoptosis (Li et al., 2022), hepatic fibrosis (Hamidipoor et al., 2015), and cardiac injury (Yang et al., 2022). These studies show clear toxicity thresholds at much higher levels than those used in our experiment. For endosulfan, multiple studies in Japanese quail have administered dietary concentrations between 5–50 mg/kg feed to assess chronic sub-lethal toxicity, documenting progressive hematological, hepatic, and reproductive alterations without mortality (Hussain et al., 2020; Qamar et al., 2012). Additional quail studies have used dietary admixtures at 100–1000 ppm to evaluate long‑term reproductive and developmental outcomes (Prakash et al., 2009). Our doses fall well within the lower spectrum of published quail dietary exposures and are consistent with established practices for assessing chronic pesticide toxicity in birds. Consequently, the selected low and high doses encompass both environmentally realistic background concentrations and a margin below known sub‑lethal thresholds, ensuring that any observed adverse effects are relevant to real‑world contamination scenarios.

**References:**

Oh, K. Y., Choi, G. H., Bae, J. Y., Lee, D. Y., Lee, S. W., & Kim, J. H. (2020). Effect of soil organic matter content on plant uptake factor of ginseng for endosulfan. *Journal of Applied Biological Chemistry*, 63(4), 401-406.

Hashmi, T. A., Qureshi, R., Tipre, D., & Menon, S. (2019). Investigation of pesticide residues in water, sediments and fish samples from Tapi River, India as a case study and its forensic significance. Forensic Science, Toxicology & Environmental Chemistry, 1-10. <https://doi.org/10.1080/15275922.2019.1693441>

Zardosht, Z., Khosravani, F., Rezaei, S., Ghaderi, S., & Hassani, G. (2023). The impact of two insecticides on the pollutant cycle and quality of surface and groundwater resources in the irrigated lands of Yasuj, Iran. Heliyon, 9(6), e17636. <https://doi.org/10.1016/j.heliyon.2023.e17636>

**Dose Formulation of Endosulfan and deltamethrin concentrations**

| **Endosulfan** | | **Deltamethrin** | |
| --- | --- | --- | --- |
| **High Dose** | **Low Dose** | **High Dose** | **Low Dose** |
| 1.5 mg/kg  Wt. of Bird:120 mg  No. of Groups:4×4  No. of Birds: 16  Trial: 21 Days  Total High Dose:  1.5×120/1000=  0.18×16×21=  **60.48 mg** | 0.03 mg/kg  Wt. of Bird:120 mg  No. of Groups:4×4  No. of Birds: 16  Trial: 21 Days  Total Low Dose:  0.03×120/1000=  0.0036×16×21=  **1.2096 mg** | 0.50 mg/kg  Wt. of Bird:  120 mg  No. of  Groups:4×3  No. of Birds: 12 Trial: 21 Days  Total High  Dose:  0.50×120/1000= 0.06×12×21=  **15.12mg** | 0.25 mg/kg  Wt. of Bird:  120 mg  No. of  Groups:4×3  No. of Birds: 12 Trial: 21 Days  Total Low  Dose:  0.25×120/1000= 0.03×12×21=  **7.56mg** |
| **Total Dose (H+L) =**  **60.48+1.2096=61.6896mg** | | **Total Dose (H+L) =**  **15.12+7.56=22.68mg** | |

**The selection of Study Duration**: Selection of Study Time Duration: A 3‑week treatment period was selected because Japanese quails exhibit rapid physiological turnover and respond measurably to dietary and toxicant exposures within short sub‑chronic windows. Previous quail studies have successfully detected metabolic (Ahmad et al., 2023), hematological (Moraes et al., 2021), and histopathological effects (Mahmoud et al., 2024), within 3 – 5 weeks of treatment. Pesticide toxicity has produced significant changes even within 2 weeks (Mahmoud et al., 2024). Therefore, a 3‑week duration provides a scientifically appropriate and validated timeframe to observe hepatic and neurobehavioral alterations in quails.”

**References**

de Moraes, L. R., Delicato, M. E. A., da Silva Cruz, A., da Silva, H. T. F. N. P., de Vasconcelos Alves, C. V. B., Campos, D. B., Saraiva, E. P., da Costa, F. P., & Guerra, R. R. (2021). Methionine supplementing effects on intestine, liver and uterus morphology, and on positivity and expression of Calbindin-D28k and TRPV6 epithelial calcium carriers in laying quail in thermoneutral conditions and under thermal stress. PLoS ONE, 16(1), e0245615. <https://doi.org/10.1371/journal.pone.0245615>

Mahmoud, U.T., El-Reda, G.A., Ali, F.A.Z. et al. Plasma activated water effects on behavior, performance, carcass quality, biochemical changes, and histopathological alterations in quail. BMC Vet Res 20, 391 (2024). https://doi.org/10.1186/s12917-024-04231-4

oral gavage

**Gauges used for feeding:** Oral gavage was used in this study, as shown in the picture below.

**Supplementary Fig. S1.** Insecticide mixture solutions and dosing gauges used for oral administration to quails.
